# Promoting the advancement of otology and audiology: a history of the Thomas Wickham-Jones (TWJ) Foundation

**DOI:** 10.1017/S0022215124001191

**Published:** 2025-01

**Authors:** M Wickham-Jones, M Bailey

**Affiliations:** 1 Professor of Political Science, School of Sociology, Politics and International Studies, University of Bristol, Bristol, UK; 2 Executive Chairman of the TWJ Foundation and formerly Consultant Otolaryngologist, Great Ormond Street Hospital for Children, London, UK

**Keywords:** otology, audiology, fellowships, scholarships, historical aspects

## Abstract

**Objectives:**

For 50 years, the Thomas Wickham-Jones (TWJ) Foundation has promoted the advancement of otology and audiology in the UK and Republic of Ireland through a series of overseas Fellowships and other grants.

**Methods:**

The paper examines the history of the Foundation since its establishment in 1974, drawing upon the Foundation's archives and personal recollections. The analysis is located within a framework concerning the factors that shape the success or failure of a foundation including vision, strategy, information, leadership and finance.

**Results:**

The activities of the TWJ Foundation are charted over five decades, and the governance of the Foundation is detailed. Particular attention is given to the Major Fellowships offered, at first in North America, and to their subsequent development into the Foundation's current rotation.

**Conclusion:**

The paper offers an assessment of the TWJ Foundation's impact on the training of otologists in the British Isles and concludes with a brief self-reflective analysis.

## Introduction

In 1971, as a student at the University of Edinburgh, Gordon Brown needed emergency surgery on a detached retina to save his remaining good eye. The consultant deferred to a young surgeon, Hector Chawla, who promptly delayed his holiday to operate successfully on the future British prime minister. Years later Brown recalled, ‘Hector had recently returned from a year in America and I was blessed to be the beneficiary of his newly acquired techniques’.^1^ Fifty years on from its founding, the Thomas Wickham-Jones (TWJ) Foundation promotes similar insight into the benefits of disseminating innovative surgical practices as a central aspect of its raison d'être. In this brief historical article, we outline an account of the Foundation and its development as well as offering a self-reflective assessment of its impact (an essential aspect of learning through experience).

There is a large and comprehensive literature on the nature of the charitable sector in modern societies. Relatively little of it, however, directly addresses the question of what determines a foundation's strengths and weaknesses. Rather, much of the literature focuses on the normative dimension to such activities and the motivations for support. In their 2016 account, however, Ruth Gripper and Iona Joy identify several factors shaping success or failure, including vision, strategy, information, leadership, and finance.^2^ We return to these issues in our conclusion.

In early 1974, Lillian Higgs (1881–1979), then in her nineties, established the TWJ Foundation together with Patrick Jobson to commemorate her father, Thomas Wickham-Jones (“TWJ”), a London wharfinger. Pat Jobson (1913–1998), an otologist based in Guildford who was married to her niece, Gulie, gave the new charity a purpose and was its founding chair: the deed gave broad objectives ‘to promote the sciences of otology and audiology’. Such activities might include, it noted, education, travel, research and support for universities. Two other trustees joined Pat Jobson at the Foundation's first meeting in April 1974 at the Royal Society of Medicine: David Wright, another Guildford otologist, then at the start of his career, and Charles Wickham-Jones (1927–2017), a family representative living in North-East England. David Wright, in effect, subsequently designed the central means by which the Foundation would secure its goals.

Lillian Higgs actually helped to found two charities, the Lillian Higgs Personal Charitable Trust and the TWJ Foundation. Following discussions with David Wright and seeing the opportunity to start something new, Pat Jobson mapped out the scope for an otological and audiological charity. Working together at the Royal Surrey County Hospital, the two discussed informally the possibility of a foundation. That Thomas Wickham-Jones was deaf (and, by all accounts, pretty crochety with it), as was Lillian Higgs (who was not crochety at all), offered some sort of symmetry to the proposal. The Higgs Charitable Trust, as it became known, was and remains the single biggest benefactor of the TWJ Foundation with generous grants over the years. At its establishment, the TWJ Foundation had almost no capital endowment of its own and has built up funds relatively slowly over the decades. Lillian Higgs played no formal or legal part in the TWJ Foundation: she is not mentioned in its deeds (it was created by Pat and Gulie Jobson). However, Pat Jobson wrote regularly to her with accounts of its meetings, and she took a close personal interest in its Fellowships during those early years, as did her son, Tom Higgs.

## Methods

This paper examines the history of the Foundation since its establishment in 1974, drawing upon the Foundation's archives and personal recollections. The analysis is located within a framework concerning the factors that shape the success or failure of a foundation including vision, strategy, information, leadership and finance.

## Results

## Finding a format: the early years of the TWJ Foundation

One matter for the Foundation was how to attain its objectives, broad as they were. But underlying such breadth was a relatively simple premise, shared by the original trustees and Lillian Higgs: a belief than an effective way to advance otology and audiology was through education, training and travel, in combination with each other. They held a conviction that exposure to arrangements elsewhere, and the consequential broadening experience, would enhance and improve an individual's abilities. Part of the rationale was to send fellows out to centres of excellence where award-holders would learn new techniques not yet practised within the British National Health Service, at the same time as providing them with research opportunities. Going abroad meant, David Wright later recalled, that ‘you had the opportunity to see how people organised things and ran their units’ (D Wright, interview with M Wickham-Jones, November 2023). Early on, Pat Jobson wrote to Tom Higgs, ‘Our plan is to spread skills in the surgical relief of deafness throughout the UK by offering grants to enable young specialists to attend centres at home and abroad where special techniques can be learnt’ (P Jobson, letter to T Higgs, 25 April 1974). Nearly 20 years later, he put it as, ‘Our underlying purpose is to improve the training of the young otologist’ (P Jobson, letter to M Thorne, 27 February 1992).

In September 1974, the trustees considered the first round of applications (16 in total) for what became known as TWJ Otological Travelling Fellowships. They awarded two to go to Los Angeles the following January. They also made two further grants, one to go to Europe and another to study cochlear implants in the USA. These first Travelling Fellowships were quite short (around three weeks) and by February, the fellows were back and reporting on their visits: ‘It was evident that they had both gained enormously from their visit and were already putting into action some of the methods they had seen’ (TWJ Foundation minutes, 7 February 1975). Pat Jobson told Lillian Higgs, ‘They were absolutely full of enthusiasm and delighted with all they had done and seen. One had particularly gained from seeing new surgical techniques, which he had already started copying’ (P Jobson, letter to L Higgs, 10 February 1975). At meetings in 1975, the trustees made further awards for courses and research in the USA and Europe. They also agreed to help the Royal Society of Medicine (RSM) fund overseas speakers. A plan to send fellows to a temporal bone dissection course in Los Angeles proved to be too expensive and was soon dropped.

These early experiences set a pattern for the following few years of the TWJ Foundation with awards focused on informal visits to overseas departments and some training courses. At this stage, the trustees could be quite proactive. By rotating grants, they sought to target awards on a regional basis throughout the UK: ‘We also want to try and spread progress throughout the UK and not just the big centres' (TWJ Foundation minutes, 25 April 1975). On one occasion, having decided that there should be a British representative at a conference organised by Professor Ugo Fisch in Zurich, they approached a consultant at the Royal Free Hospital and asked him to attend.

## The development of Major Fellowships

In the late 1970s, the Foundation reoriented its activities, launching more formal and established annual Fellowships. The focus remained on North America: as Pat Jobson wrote to Lillian Higgs, ‘We all feel that the most benefit has been by sending consultants to America… America seems to stand out’ (P Jobson, letter to L Higgs, 28 March 1978). David Wright, as trustee, took the lead in negotiating the arrangements for a new programme aimed at UK-based senior registrars.

Back in the late 1960s, Sir Donald Harrison, professor at the Institute of Laryngology and Otology, had told David Wright, who had just completed his training, that there was an ENT vacancy at the University of the West Indies, following Kenneth McNeil's appointment as the Jamaican Minister of Health. Around three weeks later, Wright was in charge of a department at the hospital, covering the whole of the West Indies if necessary. He spent the next year working at Kingston Public Hospital as well as the university. McNeil was well-known amongst senior surgeons in the USA, and, during the year around 10 of them came over to Jamaica for a week-long closed workshop at the university.^3^ Wright built up relations with these American surgeons, who comprised some of the founding figures of modern ENT, senior professors who were breaking new ground. Subsequently, some invited Wright over to the USA and, before returning to the UK, he spent time visiting American centres to observe their work and surgery. In a sense, the TWJ Foundation was about encouraging trainees to do something similar.

Having maintained those links in North America, in the 1970s Wright developed some of them into formal Fellowships, starting with San Fransisco in 1978, working on cochlear implants (a year earlier, a TWJ fellow had spent four months in Boston). Over the next few years, Wright was able to launch further Fellowships: one in Toronto and one in Michigan, where the focus was on surgery of the ear, especially of the petrous temporal bone and the skull base (the first holder was Martin Bailey, chair of the Foundation at the time of writing).^4^ These Fellowships lasted between 6 and 12 months and included a large research component. In the case of Toronto, the Foundation met half the cost and the hosting university covered the other half.

These Major Fellowships replaced the informal travelling ones and formed a central focus for the Foundation's activities over the next decade, absorbing the bulk of its expenditure. As David Wright and subsequently other trustees built up relationships elsewhere, they negotiated new arrangements with other centres (see Table 1). A Fellowship in St. Louis lasted only one year (in 1986), as did one in Cincinnati (in 2003). In the early 1990s, the Foundation added a Fellowship in South Africa at the Groote Schuur Hospital in Cape Town: with relatively straightforward registration, it offered more clinically oriented work, and local financial support defrayed the cost to the Foundation.

**Table 1.** Major TWJ Fellowships to 2017

Location	Start	End	Host*	Location	No. of fellows
San Francisco	1978	2001	Robert Schindler and Michael Merzenich	Coleman/Epstein Laboratory	21
Toronto	1980	Ongoing	Michael Hawke, Julian Nedzelski and John Rutka	Toronto General Hospital, University of Toronto	36
Ann Arbor, Michigan	1981	2003	Malcolm Graham, Steven Telian, Merle Lawrence and Josef Miller	Kresge Institute, University of Michigan Medical Center	20
St. Louis	1986	1986	John Frederickson	Washington University	1
Cape Town	1993	2001	Sean Sellars	Groote Schuur Hospital	10
Harvard and Boston	1977, 2001	2003	Joseph Nadol and Saumil Merchant	Eaton-Peabody Research Lab	3
Cincinnati	2003	2003	Myles Pensak	University of Cincinnati	1
Melbourne	2004	2007	Robert Briggs and Robert Shepherd	The Royal Victorian Eye and Ear Hospital & Bionic Ear Institute	5
Perth	2004	2017	Marcus Atlas, Gunesh Rajan, Harvey Coates and Steve Rodrigues	University of Western Australia	6
Sydney	2004	2009	Paul Fagan	St Vincent's Hospital	3

* This table endeavours to list the main hosts: of course, others assisted in supporting the Fellowships over the years.

By the 1990s, there was less interest amongst applicants for the American Fellowships: they could be expensive, had a heavy research focus, and it had become prohibitively difficult to obtain a medical licence for clinical practice in the USA. Currency fluctuations between sterling and the dollar were a particular concern: occasionally the trustees had to consider suspending a Fellowship for a year. There were other substantive issues as well: by 1998, the trustees concluded, that ‘there was no need for trainees to learn about cochlear implants as there are now adequate opportunities for training in the UK’ (TWJ Foundation minutes, 28 April 1998). Accordingly, in the 2000s, the Foundation negotiated a new set of fellowships (although Toronto continued with a brief break), focused on Canada, Australia and New Zealand. On occasion, potential hosts approached the TWJ Foundation but were unable to finalise a fellowship, sometimes due to lack of funds on the part of the Foundation. At the time of writing, the result is a rotating programme of fellowships (see Table 2): there is a TWJ Fellow in each centre every two or three years, leaving the host institution free to appoint another international or local Fellow in the non-TWJ years.

**Table 2.** Current rotation of TWJ Fellowships as of 2023

Location	Start	Host	Location	No. of fellows
Auckland, New Zealand	2016	Michel Neeff	Auckland City Hospital & Starship Children's Hospital	4
Halifax, Canada	2017	David Morris & Nael Shoman	QEII Health Sciences Centre	2
Toronto, Canada	2019	John Rutka & Ambrose Lee	Toronto General Hospital	1
Vancouver, Canada	2021	Brian Westerberg & Jane Lea	St Paul's Hospital	1
Cambridge, UK	2021	Manohar Bance & colleagues	Addenbrooke's Hospital	1
(Toronto recommenced after a brief break)				

## Short Fellowships and other awards

The trustees did not confine themselves to these Major Fellowships. From its early years, the Foundation also funded Short Fellowships, which had a week of clinical observation and personal instruction attached to a European training course. In 1975, the TWJ Foundation made awards for the Bordeaux course run by Michel Portmann, as well as to go to Zurich to study techniques of surgery of the inner ear and internal auditory meatus with Ugo Fisch. Njjmegen and Antwerp followed some years later. During the 1990s, Jean Bernard Causse, based in Béziers, was especially supportive of the Foundation, offering board and lodging for two fellows each year on courses at the Clinique Jean Causse, founded by his father (Jean Bernard Causse died in 2001). In 2018, awards for these courses were named CWJ Short Fellowships in memory of Charles Wickham-Jones. In all, around 70 fellows went to Béziers during the TWJ Foundation's first five decades, with nearly 30 going to Nijmegen and around half a dozen to Antwerp. In the early 1990s, the Foundation made the first of occasional thesis grants to assist in the writing up of an otherwise completed research project for an MD, MS or PhD (TWJ Foundation minutes, 9 November 1989) (Wright wanted to promote academic scholarship in otology) (TWJ Foundation minutes, 8 December 1988).

Alongside the Fellowships and support for training courses, in its first decades the TWJ Foundation offered diverse grants, including the original ad hoc travel awards. During the 1970s and 1980s, there were also a handful of institutional grants to help with particular research projects. To support their research in cochlear implants, the Foundation met some salary costs at Guys in 1979 (for an investigation into the effect of cochlear implants), and it helped University College Hospital in 1982 with equipment (TWJ Foundation minutes, 18 May 1979 and 16 September 1982). It funded a temporal bone laboratory at the University of Wales, Cardiff, in 1980 (TWJ Foundation minutes, 28 August 1980). A few years later, the Foundation paid for computers and some salary costs at the Bristol Royal Infirmary to support research into the management of glue ear (TWJ Foundation minutes, 27 January 1984). Occasionally, the Foundation supported applications for equipment, including helping consultants obtain state-of-the-art microscopes. The TWJ Foundation also made small grants to BRINOS (Britain Nepal Otology Service), founded by Neil Weir (a TWJ award holder), to help fund surgical camps in the 1980s and 1990s.

Between 1981 and about 1994, the TWJ Foundation funded ENT journal subscriptions and binding at the Royal College of Surgeons of England library. Until 1995, the Foundation helped out with expenses for overseas speakers coming to the RSM Otology section; and each year since 2020, it has awarded a Short Fellowship to the Causse Clinic as a prize for the RSM Section of Otology Short Papers. Since 2010, the TWJ Foundation has sponsored a Keynote Lecture at each BACO Congress. In September 1982, the trustees established the Michael Cook Tinnitus Fund in memory of a Thames Valley farmer, working with his family, with a focus on audiology: it still funds the Pat Jobson Prize, which is awarded annually by the British Association of Audiovestibular Physicians (BAAP).

At times, there was a policy dimension to these activities. In 1978, the Foundation funded the publication of the Ballantyne report on cochlear implants.^5^ Earlier, it had helped finance research for the report with a symposium at Stanford University. John Ballantyne's JLO obituary noted the report's importance in facilitating the take-up of cochlear implantation by the Department of Health and Social Security.^6^ In 1990, the TWJ Foundation was responsible for publication of a BAAP policy document on the care of deaf children (TWJ Foundation minutes, 8 February 1990). The Foundation supported audiologists working on rehabilitation with cochlear implant programmes at this time with trips to Australia and the USA. The TWJ Foundation also appears to have held one share in *The Journal of Laryngology and Otology*, guaranteeing a sum of £20 in the event of failure (TWJ Foundation minutes, 7 December 1984).

The variety of awards reflected the flexible outlook of the trustees and the Foundation's broad remit. It also signified an era of higher dividends, which meant the Foundation had a higher income and more funds to dispense. However, in 2008 the financial crisis and accompanying reduction in dividends seriously impacted the Foundation's income, and the trustees were obliged to scale back the Major Fellowships. In effect, this situation shaped the Foundation's present strategy of a rolling programme of 12-month fellowships, rotating around different destinations from year to year. Since then, there has been some improvement in the Foundation's finances. Consolidation with two other family charities, the Jobson Foundation (which included the Furby Fund) in 2015 and the Frocester Trust (a Wickham-Jones charity) in 2018, improved the TWJ's capital endowment. At the same time, of course, the Major Fellowships have become more expensive, and in recent decades, they have been the main focus of TWJ awards.

## Running the TWJ Foundation

For the first 15 years of its existence, the three founding trustees ran the TWJ Foundation with minimal assistance, giving it an exceptionally flexible structure. There was no formal secretarial support until 1994 when David Wright took over from Pat Jobson as chair (Jobson passed away in 1998). The trustees met when they needed to, often simply to interview for a single fellowship. Running costs in this period were less than 1 per cent of its turnover, with few expenses: Pat Jobson handled the correspondence, any fundraising and the accounts, while David Wright negotiated arrangements for the Major Fellowships, including visiting the overseas centres.^7^ By the mid-1990s, interviews were combined, so that they took place for all fellowships on the same day each year. It was a decade before any of the trustees missed a single meeting. The typical pattern was to meet once a year at the RSM and once in Guildford. But there was quite a lot of variation: in 1987, they met four times in London; in 1991, they met twice in a fortnight. In the early 1990s, John Evans, another otolaryngologist at St. Thomas’ Hospital and Great Ormond Street Hospital for Children, became a trustee. The Foundation's otological assessors included Leslie ‘Sam’ Salmon, Robin McNab Jones, Harold Ludman and Valentine Hammond as well as Jonathan Hazell (for audiology).

By the early 2000s, it was clear that the original intention of having no more than three trustees, whilst good for flexibility, was no longer viable. One issue was that the Foundation should be able to establish and sustain close direct links with overseas centres hosting the Fellowships. It was also important that there should be otological trustees actively working within the British system as training within it evolved and developed. Accordingly, the Foundation expanded the number of medical trustees, with Martin Bailey and David Proops joining in 2001. They were joined by Shakeel Saeed in 2013, Chris Aldren and Musheer Hussain in 2018 and Emma Stapleton in 2021 (John Evans retired in December 2004, David Proops in December 2014, and Musheer Hussain in October 2024). From the late-2000s onwards, heads of overseas host departments have often participated at the interviews each year.

Since 2012, the TWJ Foundation has collaborated with another otological charity, the Graham Fraser Foundation which focuses on cochlear implantation, to hold one joint set of interviews each year. Graham Fraser was a pioneer of cochlear implantation in the UK, and had been a TWJ award holder and supporter of the Foundation. Around the same time, ENT UK took over responsibility as the TWJ Foundation's Secretariat.

## Impact

Assessing the impact of a foundation like the TWJ is by no means straightforward. Any perceived benefits from its work might have happened in any case, shaped by exogenous factors. Regardless of any award, beneficiaries might have achieved their goals and developed their potential. Moreover, working with the Foundation's archives, it is hard to track exactly how the careers of its award holders have developed, although there is some information in minutes and correspondence and through personal contact. In the late 1980s, when the cochlear implant programme took off in the UK, the consultant surgeons working on it in Nottingham and Cambridge included former TWJ fellows who had worked on implants in San Francisco. The TWJ Foundation had provided support for five of the six centres: Pat Jobson commented, ‘Our sponsorship has produced a lot of enthusiasm and has introduced to the problem a number of doctors who might otherwise [have] known very little about the subject’ (P Jobson, letter to T Higgs, around December 1989). After one grant-holder went to Scandinavia to look at hearing aids in 1990, she got a DHSS grant ‘for a unit at Great Ormond Street Hospital for Children to make it possible for children with gross cranial deformities to use hearing aids’ (TWJ Foundation minutes, 8 February 1990) (although, of course, she might have got the award anyway). In 1999, one recipient of a thesis grant wrote that completion of the project ‘would not have been possible without the support of the TWJ Foundation’ (a comment that quite often crops up in the reports regarding theses).

**•** The Thomas Wickham-Jones (TWJ) Foundation celebrates its 50th anniversary in 2024
• Little has been written about the history of the TWJ Foundation, yet a very large proportion of British otologists has benefited from a TWJ grant.
• This paper details the history and development of the TWJ Foundation and its programme of Fellowships and other activities; and its contribution to British otology and audiology
• The paper assesses the impact of the Foundation's Fellowships and offers a self-reflective analysis of the TWJ Foundation

As importantly, demand for grants and fellowships has endured. The reports submitted at the end of fellowships give a sense of the perceptions of award-holders and what they have taken away from their fellowship. With the Major and Short Fellowships, fellows conclude that they have gained experience, learnt new techniques, shared ideas, and developed their own skills. They have observed practical and innovative demonstrations, and they have noted the differences in surgical arrangements and healthcare systems across national borders. They have been able to ask questions, get support, and have received feedback. The experience has enthused them and directly shaped their practices back in the UK. One way to convey these conclusions is through their reports back to the Foundation (which can be read at https://twjfoundation.org/reports/). The following anonymous quotations, a very small but hopefully representative sample, give a flavour of these (with added emphases). From the Major Fellowships:

- ‘The multidisciplinary management of these clinics was an eye opener to me and *changed* my entire perception and understanding of managing dizzy patients. I spent a lot of time learning and improving my skills in advanced middle ear and lateral skull base surgery’ (2014).
- ‘My time in Toronto has provided me with not only a wide range of clinical and surgical skills but also a life-enriching experience I will never forget…. taught me how to master the reverse order stapedotomy. This was a new technique I had never seen but now plan to *adopt* into my future practice’ (2017).
- ‘Not only have I improved my skills, knowledge, judgement and experience dealing with complex and advanced cases, but I have *cultivated* a different clinical management approach and alternative surgical philosophy’ (2017).

Regarding Short Fellowships to Béziers, fellows have written:

- ‘The optics in theatre were awesome and allowed an excellent view of proceedings and both surgeons were happy to answer questions throughout their cases… [of one technique] This is something I am keen to *adopt* after seeing the excellent access that can be obtained’ (2018).
- ‘I came away with a wealth of newly acquired surgical knowledge and numerous small tips that I will be *applying* in my theatre’ (2017).
- ‘The Fellowship was highly enjoyable and highly educational…. I took away a great deal from my visit, things that will *influence* my practice and ultimately, I hope, improve the care that I can deliver for my patients’ (2015).

And, from Nijmegen and Antwerp:

- ‘There are innumerable tips that will *filter* into my practice as well as the general enthusiasm for otology and teaching’ (2017).
- ‘I feel that I have learnt a great deal and acquired a huge array of tips and techniques from a surgical perspective which I look forward to *implementing* in the future’ (2019).
- ‘I have already *utilised* a lot of these new techniques and ideas learned during my time in Nijmegen within my UK practice’ (2019).
- ‘I genuinely feel that I have *learnt* a new way of tackling cholesteatoma’ (2019).

Back in 1992, after visiting Portland, Oregon, one grant holder wrote to the trustees, ‘We have already begun applying this knowledge and information in our work’ (TWJ Foundation minutes, 19 January 1993). David Wright remembered that ‘from the early days people came back from their six months and they were different people’. He recalled, ‘I think what it does is help people to be better surgeons: that's basically what it is. You do have your eyes opened when you go and work somewhere else. You bring back those ideas and they start you off, they get you interested in something, and you can see how to handle patients.’

## Conclusion

In all, over the past five decades, the TWJ Foundation has made over 400 awards, including around 125 Major Fellowships, about 80 Short Fellowships, and around 50 further grants for travel. A very large proportion of UK consultant otologists have therefore been either TWJ Fellows or award-holders. Reflecting on the Foundation's experiences, two strengths appear to stand out. The first concerns the broad aims and flexibility of the Foundation. Over its first decade, the TWJ Foundation developed a clear format based around its Major Fellowships and support for Short Fellowships attached to training courses. This arrangement facilitated a strategy to secure the Foundation's overall vision. If a fellowship did not work for the Foundation and its award-holders, it could be replaced. At the same time, as far as resources have allowed, the TWJ Foundation has been able to experiment over the years in terms of its grants and awards. The second strength concerns the close relationship between the medical trustees, exercising leadership, and representatives of the Wickham-Jones family. The support of the Higgs Charitable Trust has been crucial in this regard; but so too has the capacity of the medical trustees to negotiate, sustain and adjust the Major Fellowships. In particular, Wright and Bailey's work as chairs (along with David Proops, co-chair from 2008 to 2014) helped the Foundation develop from its initial origins to its current position. A flexible structure, underpinned by medical expertise and the passionate commitment of its chairs, has greatly assisted the Foundation in the pursuit of its goals. Returning to the assessment criteria mapped out in our introduction, the TWJ Foundation has developed a clear purpose to secure its visions, making use of medical expertise through its otological trustees. Information is used as a direct feedback mechanism either to sustain or to adjust the pattern of grants and arrangements for the Fellowships. Moreover, the Foundation has worked carefully as a third sector organisation alongside the state (in the form of the National Health Service) and the Royal College of Surgeons.

One weakness, however, has been in fundraising. The TWJ Foundation remains a niche charity. It has a clearly defined interest in otology and audiology, and what has become a well-developed strategy to attempt to secure its goals; but that character may inhibit fundraising. Higher profile foundations with either less focus or apparently more immediate and urgent concerns may attract funds more easily. Accordingly, the Foundation remains dependent upon its relationship with the Higgs Charitable Trust. As Pat Jobson told Tom Higgs in December 1990, ‘Your continued support is really our life blood’. Hopefully, however, this structure means that the Foundation is well positioned to meet the challenges of the future.

When Pat Jobson and David Wright established the TWJ Foundation, there were few specialist otological surgeons in the United Kingdom. Through its Fellowships and awards, the Foundation has helped shape the development of otology as a defined sub-speciality within ENT departments in the British Isles to the great advantage of the deaf.

## Data Availability

The paper draws upon TWJ minutes and archives, kept at the ENT UK office at the Royal College of Surgeons of England, London; as well as upon personal recollections. For further information contact the corresponding author at m.wickham@bristol.ac.uk.

## References

[1] Brown G. My Life, Our Times. London: Bodley Head, 2017:48

[2] Gripper R, Joy I. What Makes a Good Charity? London: New Philanthropy Capital, 2016

[3] Bowen C. Remembering Ken McNeill. *Jamaican Gleaner* 14 December 2001

[4] Jobson P. Ten Years of the TWJ Foundation. Guildford: TWJ, 1984:5

[5] Ballantyne JC, Evans EF, Morrison AW. Electrical auditory stimulation in the management of profound hearing loss. J Laryngo Otol 1978;92(suppl 1):1–117 281445

[6] Weir N, Brown A. Obituary: John Ballantyne. J Laryngol Otol 2008;122:1137–8

[7] Wright D. The TWJ Foundation The First Twenty-Five Years. Guildford: TWJ, 1999:2

